# Large differences in catch per unit of effort between two minnow trap models

**DOI:** 10.1186/1756-0500-6-151

**Published:** 2013-04-16

**Authors:** Juha Merilä, Hanna-Kaisa Lakka, Antti Eloranta

**Affiliations:** 1 Ecological Genetics Research Unit, Department of Biosciences, University of Helsinki, Helsinki, 00014, Finland; 2 Department of Environmental Sciences, University of Helsinki, Lahti, 15140, Finland; 3 Department of Biological and Environmental Sciences, University of Jyväskylä, Jyväskylä, 40014, Finland

**Keywords:** Fishery, Trap, Catchability, CPUE, Pungitius pungitius, Stickleback, Funnel trap

## Abstract

**Background:**

Little is known about variation in catch per unit of effort (CPUE) in stickleback fisheries, or the factors explaining this variation. We investigated how nine-spined stickleback (*Pungitius pungitius*) CPUE was influenced by trap model by comparing the CPUEs of two very similar minnow trap models fished side-by-side in a paired experimental design.

**Results:**

The galvanized trap type (mean CPUE = 1.31 fish h^–1^) out-fished the black trap type (mean CPUE = 0.20 fish h^–1^) consistently, and yielded on average 81% more fish.

**Conclusions:**

The results demonstrate that small differences in trap appearance can have large impacts on CPUE. This has implications for studies designed to investigate abundance and occurrence of fish using minnow traps.

## Background

Catch per unit of effort (CPUE) is an important variable in fisheries sciences, as it provides means to monitor population size trends (e.g. [1]), relative abundance of species in different habitats and sites (e.g. [2]), as well as to compare efficiency of different fishing gear [3-5]. It is well established that CPUE can be influenced by various environmental factors [6-10], show profound seasonal variation [11] and be sensitive to the type of fishing gear used [4,5,12,13]. For instance, Guy et al. [3] compared CPUE for white crappies (*Pomoxis annularis*) as estimated from gill and trap nets, and found little correspondence between the estimates obtained with different gear across different reservoirs and years.

Understandably, factors influencing the CPUE have been mainly studied in species of commercial interest. Less effort has been put towards studying factors that influence CPUE in species such as sticklebacks (Gasterostidae), which are popular models for ecological and evolutionary biology research (e.g. [14,15]) yet hold little or no commercial interest. Recently, influence of trap type and use of bait on CPUE of nine-spined sticklebacks (*Pungitius pungitius*) was investigated [16], and clear trap type and bait effects were revealed. However, the above mentioned study is thus far the only quantitative study focused on factors influencing CPUE in sticklebacks, and was restricted to two collapsible trap types.

The aim of this study was to test whether two commonly used and superficially similar metallic minnow trap types differ in their CPUE when catching nine-spined sticklebacks.

## Methods

**Figure 1:**
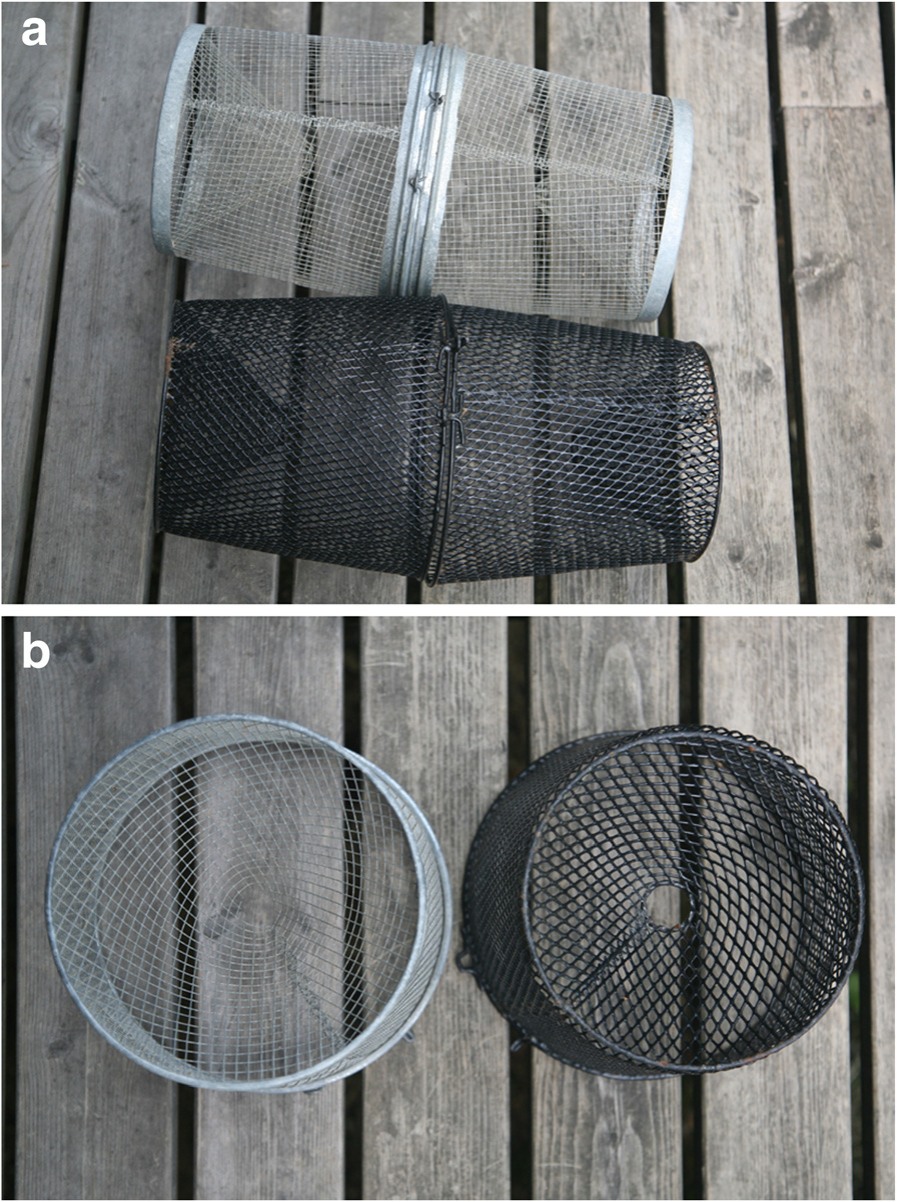
**The two minnow trap types used. (a)** View from above and **(b)** from trap entrance.

The trapping was conducted in Rytilampi (ca. 66°23^′^07″N, 29°18^′^42″E), which is an isolated pond (surface area = 4.98 ha) with maximum depth of ca. 5.1 m. The two different minnow trap types, both manufactured by Frabill (Jackson, Wisconsin, USA) are pictured in Figure 1. One of the trap types was a galvanized funnel shaped trap (also known as *GEE Minnow trap*) measuring 419 mm × 229 mm (model # 1279), henceforth referred to as the *silver trap*. The other trap type (model # 1271) was similar to the silver trap in its dimensions, but had a black vinyl coating (Figure 1a), and is henceforth referred to as the *black trap*. Moreover, while the netting in the silver trap is square-shaped, that of the back trap is diamond-shape (Figure 1a). Both trap types have two round entrances (ca. 25 mm in diameter; Figure 1b) and are widely used by researchers (e.g. [7,17]) and fishermen (see Discussion). All traps were set unbaited.

Set 1: Six pairs of traps, each consisting of one silver and one black trap, were set on July 7 2012 between 20.00–20.30, and retrieved July 8 2012 between 13.00–14.00. This corresponds to an average soak time of 16.6 h (range: 16.4–16.7) and 199 trap-hours (=12 traps × 16.6 h). The traps were lowered to the bottom in a pairwise fashion at depths ranging from 2–3.8 m, and their distance from shore ranged between 5 and 50 m. When the traps were retrieved, the number of nine-spined sticklebacks in each trap was counted and recorded. Water temperature during the trapping was about 18°C at a depth of 10 cm from the surface.

Set 2: A second set, also consisting of six pairs of traps, was set on July 10 2012 between 06.15–06.35, and retrieved July 11 between 06.20–06.35. This corresponds to an average soak time of 24.0 h (range: 23.9–24.3) and 288 trap-hours. Setting was done otherwise in a similar fashion as in Set 1, but the traps were fished from the shore at depths between 0.6–1 m. The trap pairs in this set were located 5–50 m apart from each other.

The CPUE for a given trap was estimated as the number of fish caught divided by trap-specific soak time, yielding an estimate of number of fish caught per hour.

The data was analyzed using a generalized linear mixed model (GLMM) in which the number of fish caught was treated as Poisson distributed response variable, and the trap type and set (i.e. the two catching occasions) as factors. This analysis was supplemented with two non-parametric univariate tests suited for analyzing data of matched-pair nature. First, a Wilcoxon Signed Rank test was used to test whether there was a difference in CPUE between paired silver and black traps. Second, a more conservative sign test was used to the same effect. Two-tailed tests were used. All analyses were conducted using JMP statistical software (ver. 9.0.0; SAS Institute Inc.) with an Apple Macintosh platform.

### Data accessibility

All the data is given in Table 1.

**Table 1:** Number of nine-spined sticklebacks caught in each of the traps in the two sets

		**Number of fish**	
**Set**	**Trap-pair**	**Silver trap**	**Black trap**
1	1	46	2
1	2	43	17
1	3	32	5
1	4	47	0
1	5	24	8
1	6	33	7
2	7	7	1
2	8	4	0
2	9	7	0
2	10	16	0
2	11	11	0
2	12	6	1

### Ethics statement

The work described in this paper does not constitute an animal experiment in legal sense, and hence, the only required permissions were national personal fishing licenses for 2012 (possessed by all authors) and a license (# 3087/41/2011) from Metsähallitus, owner of the water body where the fishing was done.

## Results

**Figure 2:**
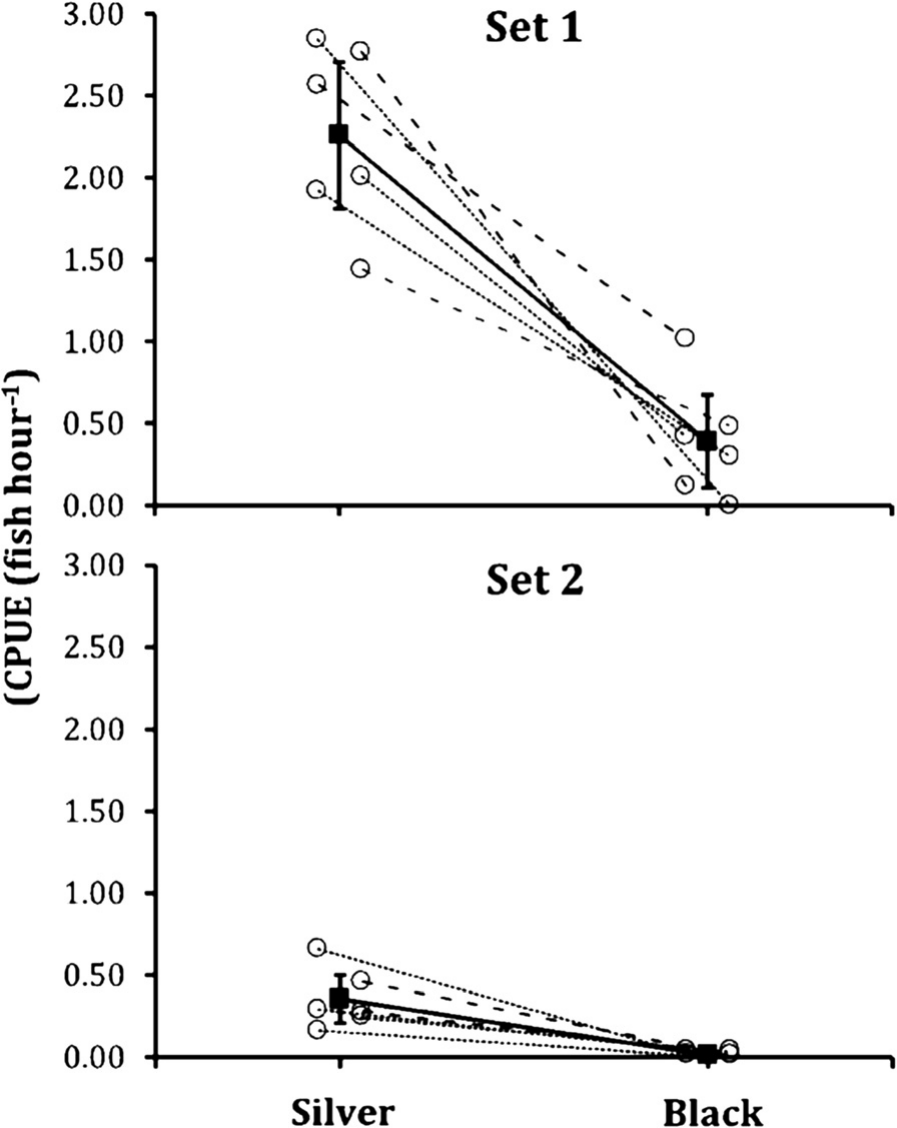
**The number of fish caught per hour (CPUE) of nine-spined sticklebacks caught from deep (Set 1) and shallow (Set 2) sites with silver- and black-colored traps.** The horizontal lines connect trap-pairs. Black boxes indicate mean ± 95% Cl CPUE for both sets. Each data point (circles) depicts CPUE in each trap (jittred for ease on interpretation).

A total of 317 fish were caught, of which 276 were from silver traps and 41 from black traps (Table 1). GLMM showed that the number of fish caught differed significantly between trap types (*χ*^*2*^ = 140.12, *P* < 0.001) and the two sets (*χ*^*2*^ = 98.09, *P* < 0.001; Figure 1). Also, the trap-type*set interaction was significant (*χ*^*2*^ = 6.04, *P* = 0.014), showing that the difference between trap types was less pronounced in the second as compared to the first set (Figure 2).

Wilcoxon Signed Rank test confirmed the GLMM results: CPUE in silver traps (mean ± S.E. = 1.31 ± 0.31) was significantly (on average 6.5 times) larger than in black traps (mean ± S.E = 0.20 ± 0.09; *S* = -39.00, *P* = 0.0005). The same conclusion was reinforced by the sign test (*M* = –6.00, *P* = 0.0005).

## Discussion

The results of this study clearly demonstrate that the silver traps out-fished the black traps, with a large margin. This was not entirely unexpected, as earlier experiences in trapping three-spined sticklebacks (*Gasterosteus aculeatus*) with these same traps in northern Finland have left an (unquantified) impression that the silver traps are more effective than the black traps. Similar testimonies are also available from North America: several customer reviews (http://www.cabelas.com) of these traps state that silver traps catch more minnows, shiners and crawfish than black traps (e.g. Anonymous, March 10 2010: “Side by side, I’ll have two-three times more minnows in the galvanized trap vs. black coated trap”; Chrisfromiowa, July 3 2007: “…side by side the black traps against the steel and steel always pull in more bait”). Hence, the better catch rate of silver over black traps appears to be a general – albeit as yet poorly quantified – phenomenon across different target species, habitats and geographic localities.

Why do the silver traps catch more fish than the black traps? One possible explanation is that the shiny appearance of the silver traps attracts fish for some reason, or that fish caught within silver traps escape (cf. [18]) less frequently than fish caught within black traps. Another possibility is that the dark and shadier appearance of the black traps repels fish. In fact, although the basic dimensions of the two trap types are the same, the vinyl coating of the black traps, in combination with their diamond shaped meshing, renders the black traps more “closed” and shady (Figure 1). This might influence the entry and/or retention rate of fish, and thereby also catchability. Differences in catchability of square and diamond shaped meshing have earlier been reported in the context of pelagic herring (*Clupea harengus*) trawling [19].

Attraction by conspecifics might also play a role in the marked difference in the CPUE performance between trap types. If fish within silver traps are more visible to conspecific than fish in black traps, this might increase the catchability of the silver traps. Naturally, the electrochemical characteristics of the two trap types are also likely to differ, but the significance that this might bear on catches remains to be investigated. Whatever the reason for the difference, it is an undisputed fact that silver traps outperform black traps in catching nine-spined sticklebacks, and possibly, also many other species of fish.

We observed that the CPUE for both trap types was markedly different between the two sets. This difference can owe to various factors, including differences in trapping location, depth, temperature and weather conditions among the two sets. Also the shorter soak time of the first as compared to second set might explain the higher CPUE in the first set (cf. [20]). Yet, in spite of these differences, the relative performance of the trap types remained the same, suggesting that the trap type rather than other circumstances may be more important determinants of the CPUE. However, as habitat complexity and predation risk are known to influence minnow trap catches [17], further experiments in different contexts (e.g. in turbid water or under predation risk) are needed to evaluate whether the results of this study can be generalized to other contexts too.

It is well established that heterogeneity in fishing methods and gear can create heterogeneity and even bias in studies seeking to estimate abundance or even distribution of particular fish species (e.g. [4,5,12,13]). Our finding that trap-type can have a large impact on CPUE of nine-spined sticklebacks adds to this evidence, and has implications for studies aiming to establish occurrence and abundance of fishes. For instance, efforts have been undertaken to map the presence or absence of nine-spined sticklebacks in large number of small lakes and ponds in Finland using foldable minnow traps [16]. In most cases presence of the species could not be established, but the surveys used trap types which were estimated to yield CPUEs in order of 0.23 fish h^-1^ in the same pond as used in this study [16]. This value corresponds quite well with the value obtained for “black” traps in the present study (0.20), suggesting that the surveys might have easily overlooked presence of nine-spined sticklebacks in low-density locations. Hence, together with earlier results, the results emphasize the need for careful selection and standardization of fishing gear in both quantitative (i.e. abundance) and qualitative (i.e. distribution) studies.

## Conclusions

In practical terms, the results of this study deliver at least two clear messages for researchers wanting to catch sticklebacks. First, the silver traps are likely to yield better catches than the black traps. Second, if different types of traps are used, it will be important to control for variation in CPUE due to trap type. In the future, studies focusing on behavioral mechanisms underlying differences in catchability by different trap types could provide interesting insights on fundamental biological questions about factors influencing animal decision making.

## Competing interests

The authors declare that they have no competing interests.

## Authors’ contributions

JM conceived the study. JM, AE, H-KL carried out the field work. JM analyzed the data. JM, AE, H-KL all contributed to writing the manuscript. All authors read and approved the final manuscript.
